# Metal-Based Drug–DNA Interactions and Analytical Determination Methods

**DOI:** 10.3390/molecules29184361

**Published:** 2024-09-13

**Authors:** Adriana Corina Hangan, Luminița Simona Oprean, Lucia Dican, Lucia Maria Procopciuc, Bogdan Sevastre, Roxana Liana Lucaciu

**Affiliations:** 1 Department of Inorganic Chemistry, Faculty of Pharmacy, “Iuliu-Hațieganu” University of Medicine and Pharmacy, 400012 Cluj-Napoca, Romania; adriana.hangan@umfcluj.ro (A.C.H.); loprean@umfcluj.ro (L.S.O.); 2 Department of Medical Biochemistry, Faculty of Medicine, “Iuliu-Hațieganu” University of Medicine and Pharmacy, 400012 Cluj-Napoca, Romania; lprocopciuc@umfcluj.ro; 3 Clinic Department, Faculty of Veterinary Medicine, University of Agricultural Science and Veterinary Medicine, 400372 Cluj-Napoca, Romania; bogdan.sevastre@usamvcluj.ro; 4 Department of Pharmaceutical Biochemistry and Clinical Laboratory, Faculty of Pharmacy, “Iuliu-Hațieganu” University of Medicine and Pharmacy, 400012 Cluj-Napoca, Romania; liana.lucaciu@umfcluj.ro

**Keywords:** DNA, DNA interactions, metal complexes, bioinorganic chemistry

## Abstract

DNA structure has many potential places where endogenous compounds and xenobiotics can bind. Therefore, xenobiotics bind along the sites of the nucleic acid with the aim of changing its structure, its genetic message, and, implicitly, its functions. Currently, there are several mechanisms known to be involved in DNA binding. These mechanisms are covalent and non-covalent interactions. The covalent interaction or metal base coordination is an irreversible binding and it is represented by an intra-/interstrand cross-link. The non-covalent interaction is generally a reversible binding and it is represented by intercalation between DNA base pairs, insertion, major and/or minor groove binding, and electrostatic interactions with the sugar phosphate DNA backbone. In the present review, we focus on the types of DNA–metal complex interactions (including some representative examples) and on presenting the methods currently used to study them.

## 1. Introduction

Deoxyribonucleic acid (DNA) and ribonucleic acid (RNA) are nucleic acids involved in the storage and transmission of genetic information. They are important in the development and functioning of all living organisms [1].

From the structural point of view, DNA contains a lot of phosphate and nucleobases with N and O atoms. This makes the interactions with metal cations possible and predictable. The double helix is stabilized by different cations, such as Na^+^, K^+^, and Mg^2+^, by binding to the negative charge of its backbone and by making bridges between the two strands. At the level of nucleotides, metals bind to the N and O of the nitrogenous bases, to the OH group in the deoxyribose structure, and to the phosphate group [2].

Transition metals like to coordinate with the N atoms of the nitrogenous bases, but they can also bind to the carbonyl and phosphate groups. Alkali and alkaline earth metals prefer to coordinate to the O atoms from the phosphate group and to the OH and carbonyl groups from the deoxyribose structure. The affinity of the metal cations against the DNA structure is as follows: Mg^2+^ > Co^2+^ > Ni^2+^ > Mn^2+^ > Zn^2+^ > Cd^2+^ > Cu^2+^ [3]. The binding of the metal to the nitrogenous bases seems to be sequence-dependent. Studies have shown that the metal cations Mn^2+^, Pt^2+^, Cu^2+^, and Mn^2+^ prefer to bind to guanine–cytosine-rich regions. Other metal cations, for example, Hg^2+^, like to bind to adenine–thymine-rich regions. Also, it has been demonstrated that the same nitrogenous base from the same sequence does not present the same affinity towards all metal cations [4]. So, the nitrogenous bases have different metal cation preferences. The stability of *3d* transition metal cation–nucleobase complexes is as follows: Me–guanine > Me–adenine and Me–cytosine > Me–thymine. When it comes to a physiological pH, the preferred binding sites at the nitrogenous bases level are N3 for cytosine, O4 for thymine, N1 and/or N7 for adenine, and N7 for guanine [5].

The affinity of the metal cations towards nitrogenous bases and vice versa presents the possibility of discovering new metal-based drugs that bind to different DNA regions.

Transition metal complexes interact with DNA and exhibit a broad range of applications in biology, which include antifungal [6,7], antibacterial [8,9,10,11], antimalarial [12], anticancer [13,14,15,16], anti-inflammatory [17,18], antiviral [19,20,21], and antipyretic properties. The literature has shown that the residual charge on metal ions, the coordination geometry, the ligand donor atom, and the morphology of the ligand play a key role in deciding the mode and extent of binding of complexes to DNA. In addition, there are many factors that govern the binding of metal complexes with DNA, which include the shape and size of the ligand, hydrophobicity, spin state, redox potential, and hydrogen bonding ability of the complexes. The binding studies of these small molecules with DNA are important in the design and development of new and efficient drugs targeted at DNA. The interaction of drugs with DNA is a very important feature in pharmacology that plays a crucial role in determining the drug’s mechanism of action, which is helpful for designing new molecules with more efficiency [22]. There are several methods that are used by researchers to determine the mode of interaction of metal complexes with DNA, i.e., UV-visible spectroscopy, circular dichroism, thermal denaturation, viscosity measurement, and cyclic voltammetry [23].

The main purpose of this review is to present the most common types of interactions between metal-based drugs and DNA molecules with concrete examples of metal complexes synthesized in recent years. Also, both the classic methods of determining these types of interactions and the current trends in terms of their evaluation, with their advantages and limitations, are presented. In studying the investigations of various researchers, it was determined that understanding the mode of interaction of ligands and transition metal complexes with DNA will be helpful in the development of new drugs for curing many diseases.

## 2. DNA Structure

**Figure 1:**
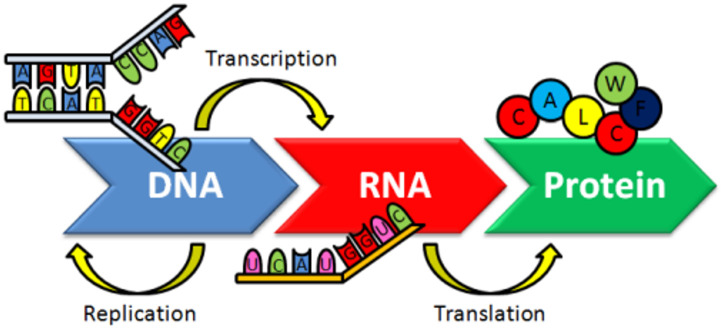
The dogma of molecular biology [24].

DNA is the genetic information carrier in living organisms. In the normal flow of biological information, upon DNA replication, the information coded in DNA is copied in messenger RNA (transcription). RNA information is then interpreted and translated into a sequence of amino acids and proteins are thus synthesized using the information in the messenger RNA as a template (translation) (Figure 1).

Genetic information is carried by genes, which are DNA sequences. Other DNA sequences participate in regulating the use of genetic information or have a structural role [24].

**Figure 2:**
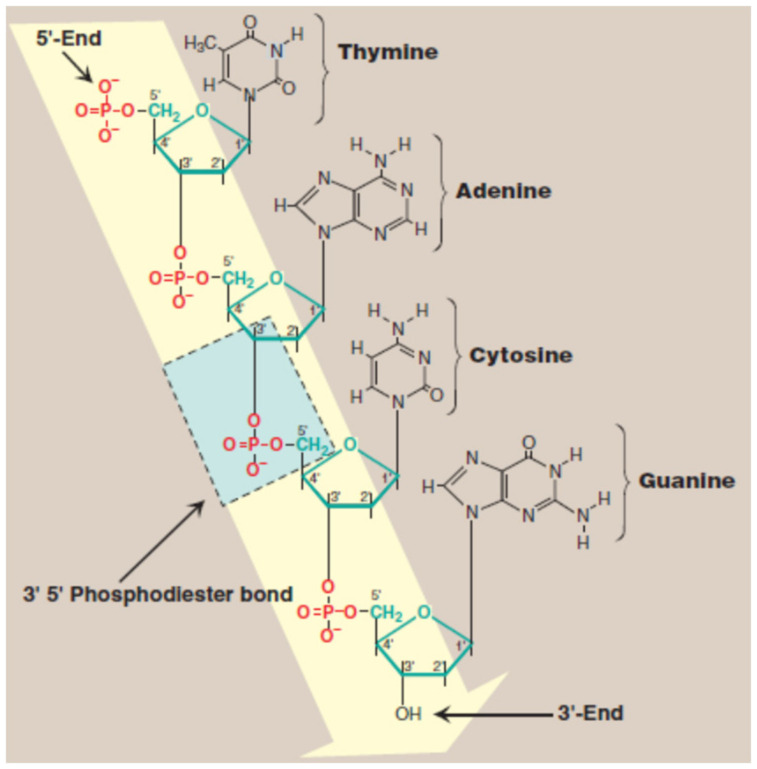
DNA structure—simplified representation. Blue indicates the 5′-3′ phosphodiester bond and yellow indicates the deoxyribose phosphate backbone [28].

Rosalind Franklin demonstrated for the first time that DNA fibers present two diffraction types: A form, the “crystalline” pattern, and B form, the “wet” pattern. She discovered the details concerning the size and shape of the DNA double helix. The way the nitrogenous bases bind inside the helix was discovered by J. Watson and F. Crick. They proved that nucleic acid has a three-dimensional structure, formed by two antiparallel polymeric chains, which contain a deoxyribose phosphate hydrophilic backbone and a hydrophobic core [25,26,27]. The simplified representation of DNA structure is presented in Figure 2 [28].

**Figure 3:**
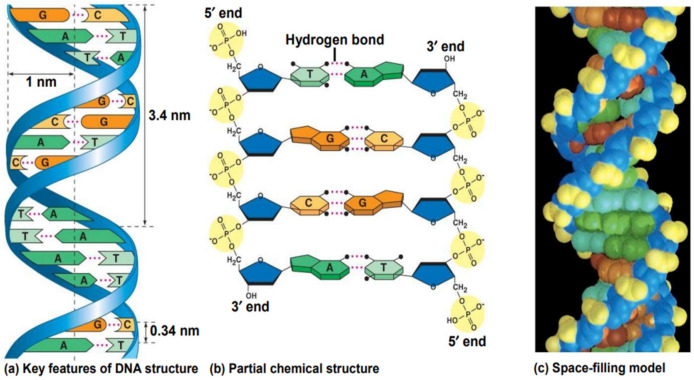
The double helix of DNA formed by hydrogen bonding between specific base pairs [30].

Hydrogen bonding between complementary base pairs, π-electron interaction between the bases stacked vertically, electrostatic forces between the negatively charged phosphate group and solvated cations, and hydration are the main factors that stabilize DNA structure (Figure 3) [28,29].

The guanine forms three hydrogen bonds with the complementary cytosine, so a high guanine–cytosine content is related to increased DNA stability. The adenine–thymine pair is linked by two hydrogen bonds, so the short helices with high adenine–thymine content show weaker interactions between the strands [31,32,33].

Two grooves are generated along the DNA chain because of the twist of the sugar phosphate backbones around the central zone of the nucleobase pairs: a major groove with a width of approximately 12 Å and a minor groove with a width of approximately 6 Å. Studies concluded that a minor groove containing more consecutive adenine residues is narrower than one with more consecutive guanine residues. Since the N-glycosidic bonds of a pair of bases are not diametrically opposed to each other, these grooves are formed. Finally, one end of the base pair is exposed to the major groove while the other end is exposed to the minor groove [34].

**Figure 4:**
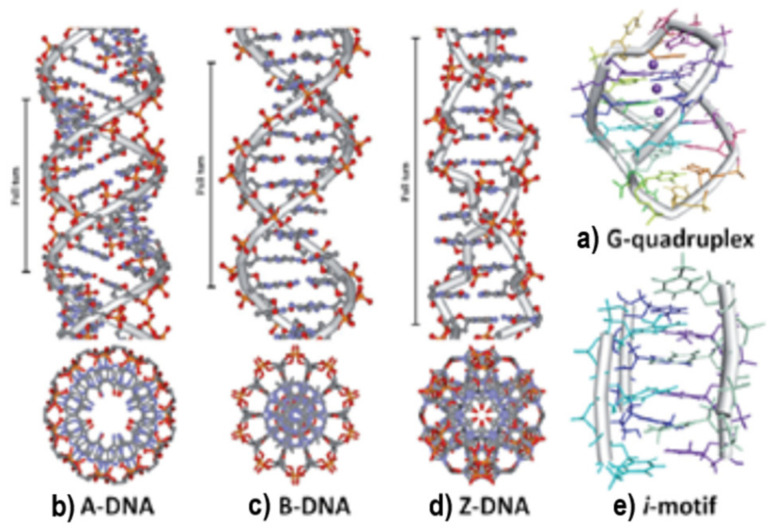
Common conformations adopted by DNA [36].

DNA adopts different conformations. They are classified as A, B, C, D, and Z forms. The primary structure of the polynucleotide determines the DNA conformation, but also environmental factors, such as hydration and ionic strength, can influence its conformation [35]. The B form of DNA is predominant in physiological conditions. The A-DNA form is a right-handed helix formed by 11 nucleobase residues on the full turn of the helix. The nucleobase pairs are tilted at 20° and migrate far from the central axis, which determines the formation of a compacted structure that contains a hollow core. The B-DNA form is also a right-handed helix, but in this structure, the size of the major groove is 10.5 Å and the minor groove is 4.8 Å (Figure 4) [36].

C- and D-DNA forms are allomorphs. Their structures are similar to the B-DNA form structure with some differences. They are obtained under low hydration conditions with Li^+^ and Na^+^ ions and their salt contents are between the salt content of A- and B-DNA forms [37].

**Figure 5:**
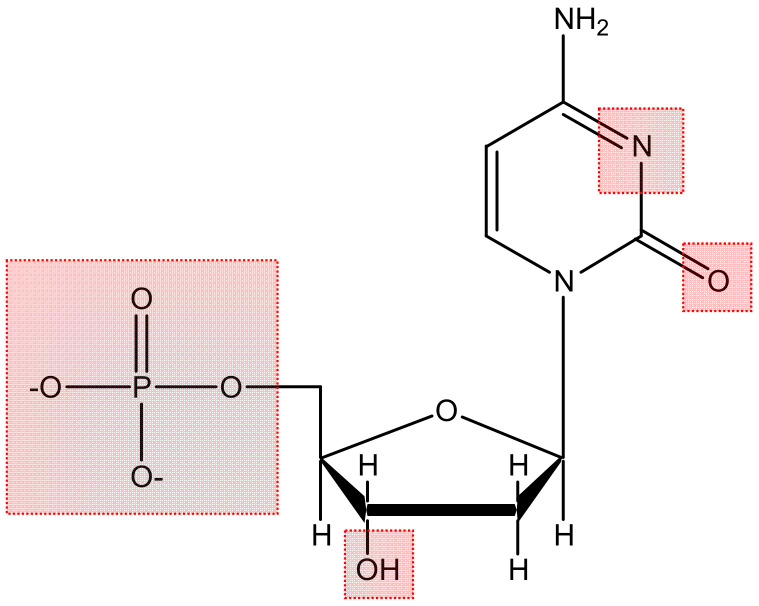
Metal-binding domains on nucleotides.

The Z-DNA form is a left-handed helix, elongated and slender in shape, which has twelve nucleobase pairs per turn, with the sugar phosphate adopting a zig-zag shape. It can be induced by the covalent bindings of different metal complexes and is adopted at high salt concentrations. The orientation of the carbohydrate groups alternates every second unit, revealing a departure from the standard mononucleotide repeating unit of the B-DNA form in favor of a dinucleotide motif. The major groove is absent because the minor groove in this structure is deeper than the one observed in the B-DNA form and expands all the way down to the axis of the molecule (Figure 5) [36].

DNA strands can be folded differently than B-DNA, or they can make Hoogsteen nucleobase pairs with the goal of forming other DNA conformations, such as hairpin, cruciform, triplex (H-DNA), or tetraplex (G-quadruplex and *i*-motif). Hoogsteen nucleobase pairs are the unusual pairs of hydrogen bonding among nucleic bases compared with Watson–Crick nucleobase pairs. Hairpin or cruciform structures are formed by self-complementary sections of single-strand DNA, where two opposite hairpins interact. In DNA genomic studies, hairpin structures have been identified and are considered potential sites for controlling gene expression. Thus, drugs that can target hairpin or cruciform structures situated in promoter zones can modify the transcription and control gene expression [38,39]. Sequences rich in guanine can form guanine quadruplex structures. They contain π-π stacking of planar guanine tetrads, bound cyclically to each other through eight hydrogen bonds according to the Hoogsteen nucleobase pairs [40,41]. A strand rich in cytosine, at a neutral or slightly acidic pH, can form the *i*-motif structure. It is also known that cytosine-rich sequences are found in, or near, the regulatory regions of more than 40% of all genes, especially in the promoter region of oncogene and human telomeric DNA [42]. The environmental conditions, the number of cytosine nucleobases, the length of the loop, and the interacting material with DNA strands significantly affect the *i*-motif structures [43,44,45].

## 3. Types of DNA–Metal Complex Interactions

DNA structure has a large number of potential binding sites for endogenous compounds and also for xenobiotics. In order to modify DNA structure, its genetic message, and its functions, xenobiotics take advantage of the binding sites at the level of the nucleic acid molecule. They modify the DNA sequence by the addition or substitution of nucleobases and hence they affect the genetic message. Following these changes, key enzymes may be inactivated and may no longer show specificity, or protein synthesis can be inhibited [46].

In order for the compound to become a therapeutic agent, it must reach the vicinity of the DNA double helix and interact with the nucleic acid molecule. The main metal-binding domains on nucleotides (Figure 5) are the O atoms contained in the negatively charged phosphate groups, the sugar hydroxyls, and the heteroatoms (N, O) on the base units [31,47,48].

The binding of the metal to the nucleobase residues is sequence-dependent. Metal ions such as Cu^2+^, Mn^2+^, and Pt^2+^ prefer regions rich in guanine–cytosine, while Hg^2+^ binds preferentially to adenine–thymine-rich regions [3]. This makes the design of metal complexes that can bind selectively to specific DNA sequences possible [49].

**Figure 6:**
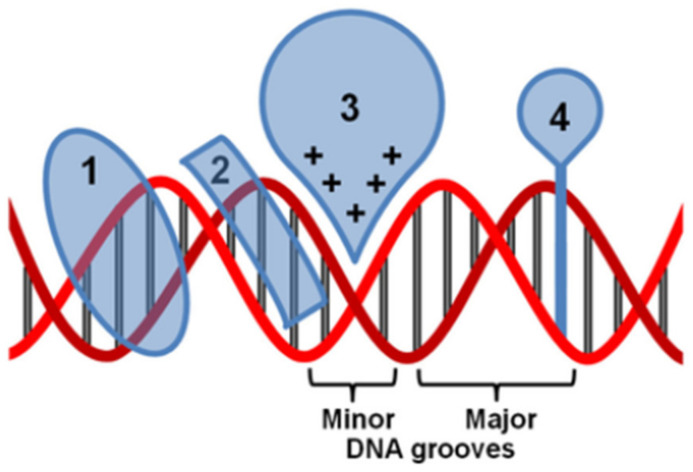
Non-covalent interaction of metal complexes with DNA. (1) A major groove binder, (2) a minor groove binder, (3) an electrostatic binding, and (4) an intercalator [50].

Currently, there are several mechanisms known to be involved in DNA binding. They are covalent and non-covalent interactions. The covalent interaction or metal base coordination is an irreversible binding represented by an intra-/interstrand cross-link. The non-covalent interaction is generally a reversible binding represented by intercalation between DNA base pairs, insertion, major and/or minor groove binding, and electrostatic interactions with the sugar phosphate DNA backbone (Figure 6) [24,50].

### 3.1. Intra-/Interstrand Cross-Link

Many anticancer drugs used nowadays in therapy exert their action by forming adducts by alkylation or inter-/intrastrand cross-linking. They covalently bind irreversibly to DNA and provoke complete inhibition of DNA functions and finally cell death.

The covalent binding to the nucleic acid in the case of metal complexes is kinetically controlled. The site and the rate of the metalation can be well modulated by a pre-association represented by the initial reversible non-covalent binding. An electrostatic interaction between DNA (a polyanion) is appropriate if the metal complex has a positive charge [48,51].

The half-sandwich-type Ru coordination compounds, with neutral face-capping macrocyclic ligands, such as 1,4,7-trithiacyclononane ([9]aneS3) or Ru–polypyridyl compounds, form adducts with guanine and its nucleoside and nucleotide derivatives. Ru–polypyridyl compounds bound strongly to DNA, both covalently and non-covalently, and were able to intercalate between nucleobases. The covalent binding of Ru-Cl-tpy compounds to L-His, L-Cys, and L-Met has also been described. The rates of the substitution reactions of Ru-[9]aneS3 and Ru-tpy complexes with biologically relevant molecules can be controlled by the choice of the inert tri- and bidentate chelating ligands, as well as by the chemical nature of the entering ligand [52].

In 1965, Rosenberg and his team demonstrated the anticancer activity of cisplatin (*cis*-dichlorodiammineplatinum(II)), the most popular anticancer drug. Following a hydrolytic step that takes place in the intracellular medium, it exerts its action by forming intra- and interstrand cross-links through the formation of covalent bonds between the nitrogen atoms from the nucleobase structures and Pt(II) ions. Both NH3 ligands remain unchanged, while both chloride ligands are replaced by the N7 atoms of adenine or guanine in order to form covalent Pt-N bonds [53].

The antitumor activity of cisplatin and analogs (oxaliplatin and carboplatin) implies a complicated cascade of reactions initiated by the formation of a DNA adduct via irreversible covalent bonding to DNA bases. The formation of the DNA adducts leads to changes in DNA interactions with proteins, generates perturbations in the cell-cycle regulation mechanisms, and can eventually lead to apoptosis [54,55].

**Figure 7:**
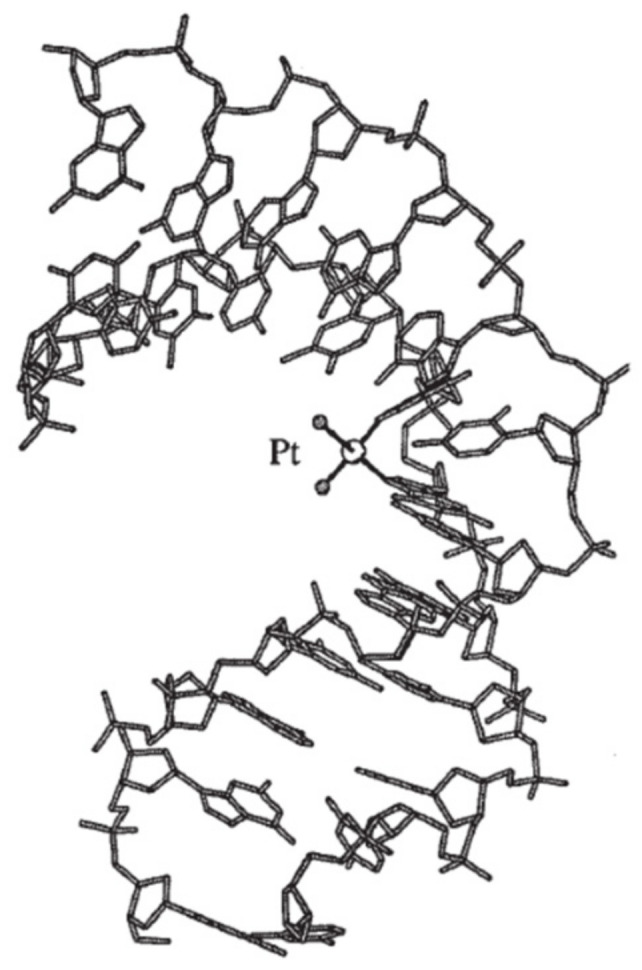
The major DNA–cisplatin adduct generating DNA deformation [58 modified].

The major site of platination in double-stranded DNA derives from intrastrand cross-links between two neighboring deoxyguanosines. Intrastrand cross-links in the adenine–guanine sequences and cross-links between two deoxyguanosines separated by a third nucleoside also occur. In the case of cisplatin and the majority of its derivatives, platination occurs via the N7 atom of the purine ring in the major groove [56,57]. Figure 7 presents the crystalin structure of the major DNA–cisplatin adduct generating DNA deformation [58].

### 3.2. Intercalators

Generally, intercalating structures are planar condensed π-deficient aromatic systems that unwind DNA in order to π-stack between two nucleobase pairs. They can be metal complexes or organic molecules. This type of interaction can be rather strong despite the fact that energy is needed in order to unwind the DNA helix and eliminate original base pair stacking interactions [59]. The stabilization of the DNA–intercalator complex takes place through van der Waals interactions between the π-electron systems of the intercalator and the heterocyclic rings of the nucleobases. Hydrogen bonds, electrostatic and hydrophobic forces, and charge-transfer interactions are also added to these interactions [60].

**Figure 8:**
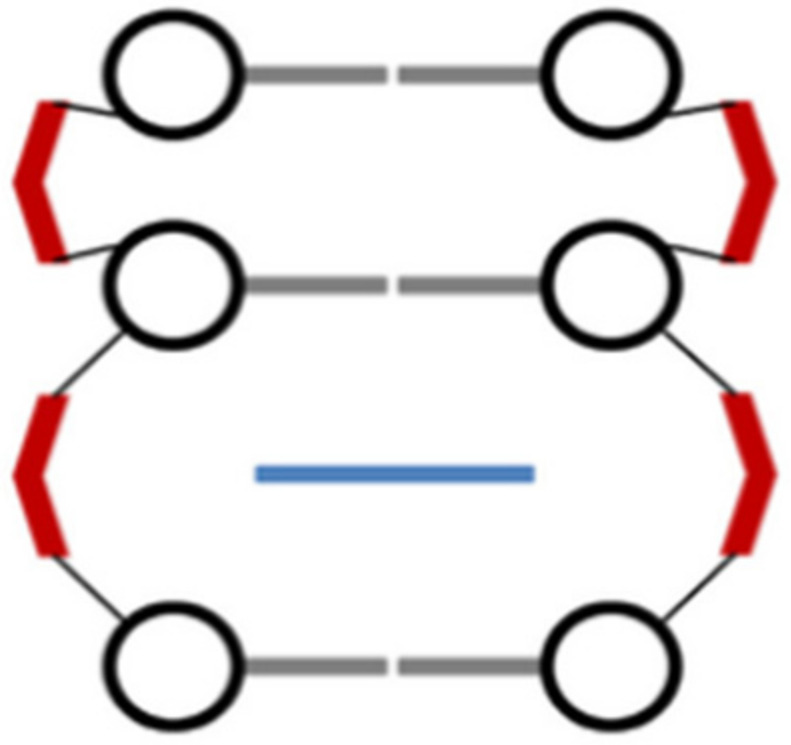
Metal complex–DNA intercalation.

Metallo-intercalators are known as metal complexes that own at least one ligand with intercalative capacity. The intercalating ligand or ligands (e.g., 1,10-phenantroline, 9,10-phenanthrenquinone diimine, dipyrido(3,2-a:2′,3′-c)phenazine, etc.), parallel positioned to the nucleobase pairs, can π-stack in the DNA helix and act as a new nucleobase pair. When it binds, the ligand behaves like an anchor for the metal complex with respect to the DNA helix and controls the orientation of the other ligands in the complex structure. Metallo-intercalators generally enter the double helix via the major groove, but intercalation via the minor groove is also possible. Intercalation requires a minimum distortion of the DNA structure, as only the opening of the phosphate angles, and not perturbations of the bases or sugar residues, are necessary. No bases are ejected from the duplex. The process results in a modification of the rise (axial distance between base pairs) and a widening of the groove at the binding site (Figure 8) [61,62].

Even though intercalation is the most common mode of interaction by which small molecules may bind to DNA, each structure may have unique binding site preferences. For instance, some like to intercalate between a 5′-pyrimidine-purine-3′ nucleobases step, while others intercalate preferentially to a 5′-purine-pyrimidine-3′ nucleobases step. Some structures have no preference for order but prefer certain base pair sequences. Intercalation lengthens, stiffens, unwinds, and stabilizes the DNA double helix [63]. Due to the fact that the metal complexes possess a rigid structure and a defined symmetry, they have the possibility to select molecular-specific DNA sequences, with the contribution of the intercalating ligand nucleobase preference and the shape and size of the auxiliary ligand. When the polyaromatic ligands have insufficient size for complete intercalation between the nucleobase pairs, or when the intercalating ligand deviates from planarity, a partial intercalation can take place [3].

The activity of mononuclear copper (II) complexes has been attributed to intercalated interactions with DNA resulting from the planar aromatic nature of the ligands [64]. Ru–polypyridyl compounds bound strongly to DNA, both covalently and non-covalently, and were able to intercalate between nucleobases [65].

### 3.3. Insertors

Insertion implies separation and displacement of a base pair. Like metallo-intercalators, metallo-insertors contain a planar aromatic ligand that enters the base stack upon DNA binding. While metallo-intercalators unwind the DNA helix and insert their planar ligand between two intact base pairs, metallo-insertors eject the bases corresponding to a single base pair. The planar ligand of the insertor therefore works as a π-stacking replacement of a nitrogenous base in the DNA structure [61].

Metallo-insertors may function as structures that recognize and selectively bind mismatched sites in DNA. DNA base mismatches (adenine–cytosine, cytosine–cytosine, etc.) occur in cells due to some errors during the replication process or after exposure to genotoxic agents and can accumulate if the cells’ defense mechanism (mismatch repair, MMR) is disabled. Thus, selective mismatch detection agents may be used as tools for the diagnosis of MMR deficiency [66].

The DNA target region of an insertor is not a unique base sequence but rather a thermodynamically destabilized region in the duplex produced by improper hydrogen bonds. As a consequence, the mismatch recognition agent would bind to any mismatched site (cytosine–cytosine, cytosine–adenine, adenine–guanine, etc.) with no selectivity for a specific sequence [61].

**Figure 9:**
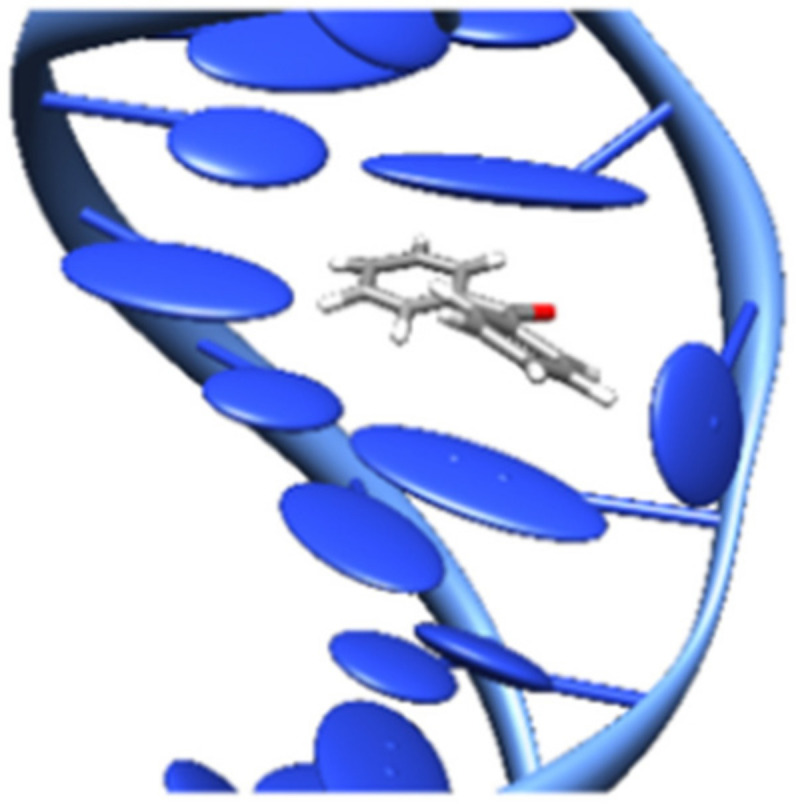
Metal complex–DNA insertion [68].

This slight destabilization has been successfully targeted through the use of rhodium metallo-insertors, which contain the sterically expansive 5,6-chrysenequinone diimine (chrysi) ligand. The chrysi ligand was designed to be larger than traditional intercalating ligands and more akin in size to a well-matched base pair, making it too bulky to simply intercalate into DNA [16]. Instead, chrysi interacts with DNA through insertion at a destabilized site. Rhodium (III) was chosen to be a substitutionally inert metal anchor for the chrysi ligand due to its photophysical properties. Rhodium complexes promote DNA strand scission as related metallo-intercalators with photoexcitation [51]. The rhodium center also anchors two ancillary ligands, which add bulk to the complexes and limit how the chrysi ligand can interact with DNA, largely preventing indiscriminate intercalation (e.g., [Rh(2,2-bipyridine)_2_(chrysi)]^3+^)_._ Another complex with the same properties is [Ru(2,2-bipyridine)_2_(dipyridophenazine)]^2+^ [67]. Figure 9 represents the metal complex–DNA insertion mode [68].

### 3.4. Major and Minor Groove Binders

The minor and the major DNA grooves, having different widths and depths, represent important binding domains on nucleic acids and exhibit structural features that may be exploited for molecular recognition of base sequences.

**Figure 10:**
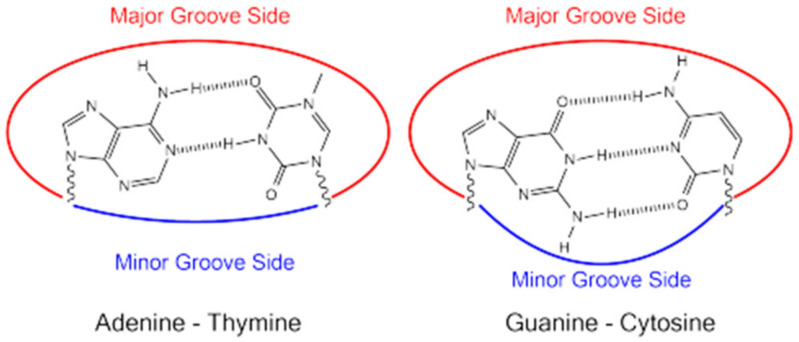
Hydrogen bonds between DNA nitrogenous bases.

Hydrogen bonds, hydrophobic, electrostatic, and van der Waals interactions are the main forces involved in DNA groove binding (Figure 10).

The interaction of DNA grooves with small molecules is also characterized by factors such as groove hydration and steric differences [69].

The structural features of the groove binders, like a matching narrow concave-shaped framework that fits in the convex DNA minor groove and the possession of electron-donating/electron-accepting groups capable of forming hydrogen bonds, allow them to interact with the nucleic acid. In general, they contain several aromatic rings, such as benzene, pyrrole, or furan, related to each other by bonds with torsional freedom. Groove binders have flexible structures that allow them to line up along the three-dimensional shape of the DNA grooves, which is the difference between groove binders and intercalators [70].

In the minor groove, hydrogen bond acceptors can serve the N3 atom of the purine structure and the O2 atom of the pyrimidine structure, and hydrogen donors can serve the amino group of the guanine structure. Within the major groove, the N7 atom of adenine–guanine, the O4 atom of thymine, and the O6 atom of guanine can serve as a hydrogen bond acceptor. The amino groups from the cytosine–adenine structure can serve as hydrogen donors. Generally, the binding of synthetic or natural heterocyclic molecules to the minor groove requires the structure to have a shape that complements the convex surface of the groove [71].

Major grooves are characterized by a great number of binding sites, they display strong binding abilities with guest molecules, and, because of their larger size, they can accommodate bulky molecules. On the other hand, minor grooves, although having fewer binding sites, often have the benefit of being free to bind small molecules.

**Figure 11:**
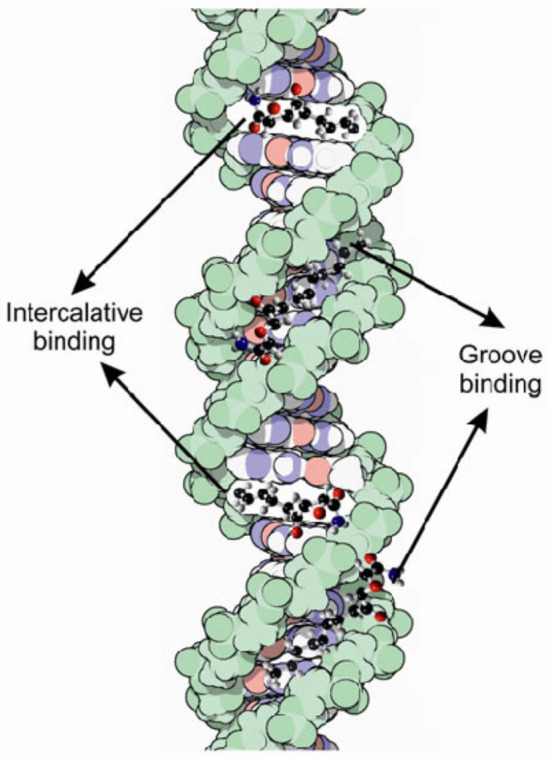
Intercalative and groove binding modes of various chemicals to DNA **[69]**.

Adenine–thymine-rich sequences are usually preferred by the groove-binding agents. This preference may be due to the better van der Waals contacts between the groove walls and the ligand, and it is also the result of the designed propensity for the electronegative pockets of adenine–thymine-rich regions. Adenine–thymine regions are narrower than guanine–cytosine ones, due to the lack of steric hindrance in the latter, represented by the C2 amino group of the guanine residue [70,72]. Figure 11 presents the intercalative and groove binding modes of various chemicals to DNA.

Generally, the binding of a Cu(II) heterocyclic complex to the minor groove requires its structure to have a shape that complements the convex surface of the groove [73].

The way in which the Cu^2+^ complex binds to the base pairs in the DNA structure is important. Firstly, the Cu^2+^ complex binds to the minor groove of the DNA. The binding takes place by the coordination of the phosphate’s oxygen atom to the metal center and is stabilized by CH···π interactions with the backbone. Then, the Watson–Crick pairing takes place when the ligands of the Cu^2+^ complex push an adenine–thymine base pair until it eventually breaks. The Cu^2+^ complex continues its action at the level of the double helix by directing both nucleobases to the major groove and then by its insertion in the newly formed cavity. The possibility of the Cu^2+^ complex being retained at the level of this cavity is due to the stacking interactions and electron depletion of the planar ligand because of charge transfer [74]. This type of interaction was found for the [Cu(4,4-dimethyl-2,2′-bipyridine)(acetylacetonate)-(H_2_O)]^+^, [Cu(phen)(acetylacetonate)-(H_2_O)]^+^, [Cu(5-methyl-phenantroline)-(acetylacetonate)(H_2_O)]^+^, and [Cu(4,7-diphenyl-phenantroline)(acetylacetonate)(H_2_O)]^+^ complexes. These Cu^2+^ complexes contain acetylacetonate as the secondary ligand [75,76]. The mixed chelate copper (II) compounds, Casiopeína IIIia (CasIIIia) and Casiopeína IIgly (CasIIgly), induce apoptosis in tumor cells by generating reactive oxygen species (ROS) and causing DNA damage. In these metal complexes, the Cu^2+^ ions bind in the minor groove in the DNA structure [77].

[Co(NH_3_)_6_]^3+^ and [Co(ethylenediamine)_3_]^3+^ are two Co^3+^ complexes that bind in the major groove at the level of guanine–cytosine sequences and can induce conformational transitions of B-DNA towards the A form [51].

### 3.5. Electrostatic Interactions with the Sugar Phosphate DNA Backbone

An external binding of electrostatic nature between a small compound and the negatively charged sugar phosphate backbone of the DNA represents non-specific interactions that usually increase the stability of a DNA–small molecule complex formed by intercalation and/or groove binding. However, they are not considered to be the main mode of binding. For metal complexes or for positively charged ligands, the electrostatic interactions are essential. There are very few examples of compounds that interact with DNA exclusively through electrostatic forces [78].

[trans(-Pt(NH_3_)_2_(NH_2_)(CH_2_)_6_(NH_3_^+^))_2_-μ-(trans-Pt(NH_3_)_2_(NH_2_)(CH_2_)_6_(NH_2_)_2_)], [Ru(2,2′-bipyridyl)_3_]^2+^ and [Ru(2,6-bis(2-pyridyl)pyridine)_2_]^2+^ are two Ru (II) polypyridyl complexes and a polynuclear platinum complex that interact exclusively with the phosphate groups in the DNA backbone [79]. Binuclear Cu^2+^ complexes, Cu_2_(OAc)_2_, have been shown to have the ability to bind to the phosphate chain of DNA through two adjacent sites [80].

In the vast majority of cases, the electrostatic interactions with the sugar phosphate DNA backbone reinforce hydrogen bonding or represent a preliminary step for the subsequent groove binding and/or intercalation.

Table 1 presents some examples of metal complexes and their type of interaction with DNA molecules.

## 4. Methods for Assessment of Metallodrug–DNA Interaction

The literature review confirmed that there are several methods used by researchers to determine the mode of interaction of metal complexes with DNA [89].

The methods used for analyzing binding phenomena can be divided into four main groups: (1) molecular spectroscopic methods that measure the spectroscopic patterns of both coordination metal complexes and DNA altered by adduct formation; (2) electrochemical techniques, mainly voltammetry, that enable the determination of changes in the redox behavior of metallic species in the presence of DNA; (3) atomic spectroscopy methods quantifying the binding via the determination of the amount of DNA-bound metal; and (4) electrophoretic methods used to assess the ability of a metal complex to cleave the DNA molecule.

### 4.1. Molecular Spectroscopy

UV-vis spectroscopy of nucleic acids is dominated by their nucleobase absorption because nucleobases have low symmetry and several heteroatom lone pairs. Transitions for individual nucleobases overlap and form a single broad and strong absorption band for the entire nucleic acid polymer, with a maximum absorbance in the range of λ = 250–280 nm. The maximum wavelength (λ_max_) depends on the adenine–thymine and guanine–cytosine content of the nucleic acid. Also, the molar extinction coefficient (ε_max_) of the nucleic acid depends on the nucleobase composition and its adopted secondary structure [90].

Absorption Spectroscopy studies suggest that metal complexes that bind with DNA through intercalation usually result in hypochromism (decrease in peak intensity) and bathochromism (increase in wavelength) due to the strong stacking interaction between the DNA nucleobase pairs and the aromatic chromophore. Hyperchromic effect (increase in peak intensity) can be assigned to external contact (electrostatic binding) or to partial uncoiling of the DNA helical structure, exposing more DNA nucleobases. The intrinsic binding constant (K_b_) value is the parameter that confirms the interaction mode of a metal complex with DNA. The K_b_ values for classical intercalators, [Ru(phen)(dppz)] (phen = 1,10-phenanthroline; dppz = dipyrido [3,2-a:2′,3′-c] phenazine) and ethidium bromide (3,8-diamino-5-ethyl-6-phenyl phenanthridium bromide) were found to be in the order of 106–107 mol L^−1^ [91,92]. Values lower than these represent an indicator of electrostatic interactions and partial intercalation.

**Figure 12:**
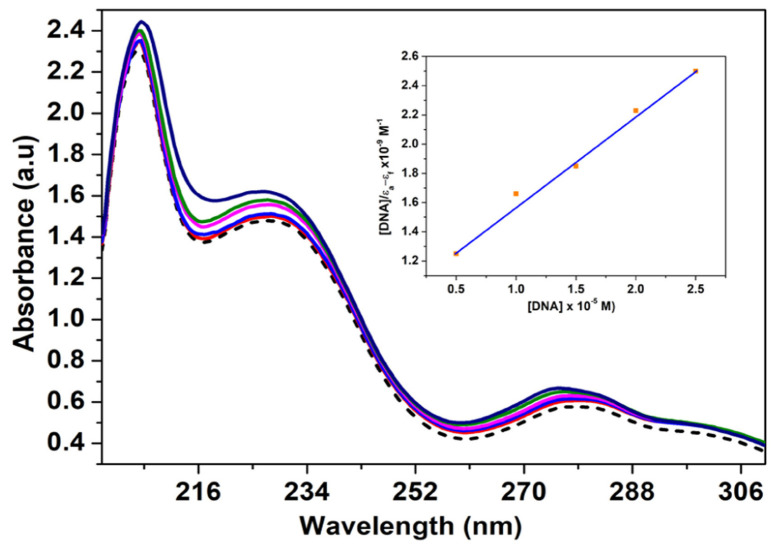
Absorption spectra of complex [Cu(N-(5-ethyl-[1,3,4]-thiadiazole-2-yl)-toluenesulfonamidate)(phenanthroline)] (20 μM) (in Tris-HCl/NaCl buffer) with increasing concentrations of calf thymus DNA. Insert shows the plot of [DNA]/(*ε*_a_ − *ε*_f_) vs. [DNA].

Ethidium bromide is a planar cationic dye. Due to its capacity to inhibit DNA biosynthesis, gene transcription, and translation, it is known as a carcinogen, mutagen, and antimicrobial agent [93]. By insertion of the phenanthridine ring between adjacent nucleobase pairs, it strongly interacts with the DNA double helix [60,94]. The interaction between anionic phosphate groups in the DNA structure and the positively charged ethidium bromide molecule is an electrostatic type. It represents the mediator for the formation of π-stacking interactions with the nucleobases, which is considered to be the main binding element [95]. The binding tendency was determined by monitoring the change in the absorbance of the complex when the concentration of CT-DNA was increased. An absorption spectrum of the Cu^2+^ complex is shown in Figure 12.

#### Fluorescence Spectroscopic Studies

Due to its high fluorescence when bound to a nucleic acid, ethidium bromide is widely used as a sensitive fluorescent probe for DNA. In a buffer solution, because of solvent quenching of a photoelectron transfer process, the free ethidium bromide molecule shows reduced emission intensity [96,97]. Because of the steric protection provided by the nucleobases to the dye molecule, when ethidium bromide binds to DNA, it shows an intensification of fluorescence [98].

The presence of a metal complex (a compound that presents affinity towards DNA) in the close vicinity of the nucleic acid determines a decrease in the emission intensity of the ethidium bromide–DNA adduct. This is accomplished by replacing ethidium bromide, accepting the excited state electron of the ethidium bromide through a photoelectron transfer mechanism, and/or modifying the conformation of the DNA molecule [97,99]. By conducting competitive fluorescence studies, the affinity of metal complexes towards DNA can be evaluated. The affinity is related to the extent of the emission intensity reduction in the ethidium bromide–DNA adduct.

By registering the emission spectra of the species upon additions of different metal complex concentrations to DNA pretreated with ethidium bromide, the quenching of the ethidium bromide–DNA adduct fluorescence was studied.

The studies based on fluorescence are limited by the fact that indirect methods were employed with a reliance on the signal from a fluorogenic reporter. Before analysis, the quenching of the reporter by the metallodrug must be examined [90].

**Figure 13:**
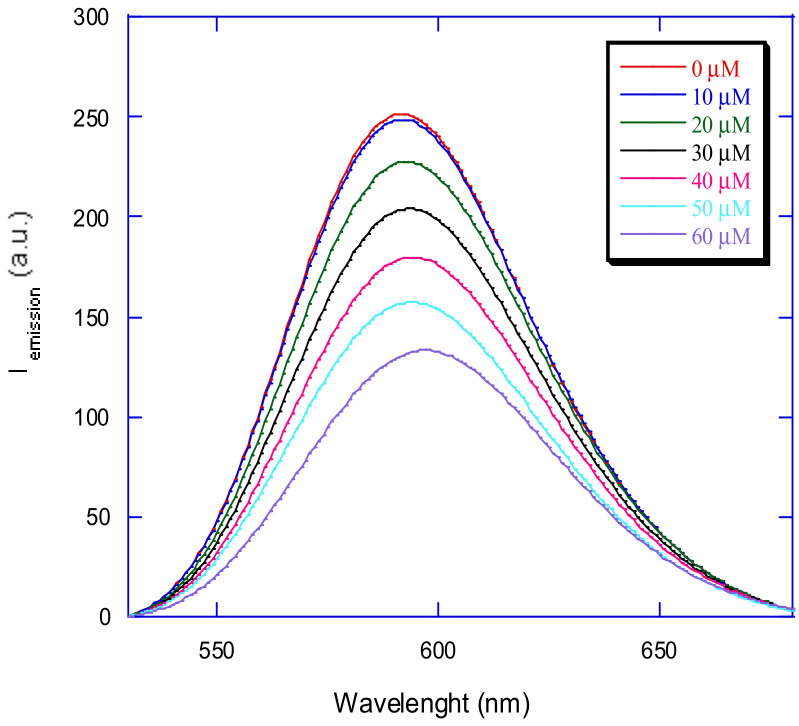
Emission spectra of ethidium bromide bound to CT-DNA (λ_ex_ = 500 nm, λ_em_ = 530–680 nm) in the absence and presence of 10, 20, 30, 40, 50, and 60 μM of [Cu(N-(5-ethyl-[1,3,4]-thiadiazole-2-yl)-toluenesulfonamidate)(phenanthroline)].

An example of emission spectra of the DNA–ethidium bromide adduct in the absence and in the presence of a Cu^2+^ complex at increasing concentrations is shown in Figure 13.

### 4.2. Electrochemical Methods

**Cyclic voltammetry** has also been used to determine CT-DNA–metal complex interactions. The binding modes can be interpreted by the variation in formal potential. Many researchers observed that a positive shift (anodic shift) in formal potential is caused by the intercalation of the cationic metal complex with the DNA double helical structure [100]. For the electrostatic interaction of the cationic metal complex with the anionic phosphate backbone of DNA, a negative shift is found [22,101]. Among different electrochemical methods, cyclic voltammetry is considered the best for the study of in vitro metal-based drug–DNA interactions in terms of changes in the redox activities. This is due to the closer resemblance between electrochemical and biological processes.

Metal–ligand coordination in metal complexes is considered most suitable for molecular recognition in biological systems and their distinct properties can be used as probes to understand and control their biological processes. The variations in the voltammetric responses of a redox-active metal complex—either in terms of changes in current or potential or both—can further be utilized to determine their binding with DNA in terms of binding constant and site size. The metal complex–DNA interaction mechanism could be determined by using either a compound-modified electrode or a DNA-modified electrode. Changes in the voltammetric curve of a metal complex in the presence of DNA concentrations depend on solvent choice, diffusion current within the site of DNA abundance, kind of ligand, charge, and the geometry of the metal center charge. These factors directly influence and control the binding equilibrium of metal complexes with DNA. Electrochemical examination of metal complexes for their binding interactions with DNA in terms of changes in redox behavior and data interpretations for kinetic and thermodynamic parameters has provided the best compliment to similar studies using physical/analytical/biological and spectroscopic techniques [102].

Cyclic voltammetry is a technique used for the evaluation of drug–DNA binding parameters. It can be assumed that the redox mechanisms that take place in the body and at the electrode level share similar principles due to the resemblance between electrochemical and biological reactions. In cyclic voltammetry, the peak potential and current intensity of the compound change if the compound interacts with nucleic acid. The changes in peak height (Ip) of the drug as a result of the addition of DNA can be used for the determination of the binding constant of the drug–DNA adduct, whereas the shift in peak potential can be exploited to ascertain the mode of interaction. The decay in the peak current of metal complexes as a result of the addition of varying concentrations of DNA was used to quantify the binding constant using the following equation:

1/[DNA] = [K (1 − A)/(1 − I/I0)] − K(1)

where *K* = binding constant, *I*_0_ = peak current of the metal complex in the absence of DNA, *I* = peak current of the metal complex in the presence of DNA, and *A* = proportionality constant. The binding constant was determined from the intercept of the plot of 1/[DNA] versus 1/(1 − *I*/*I*_0_).

**Figure 14:**
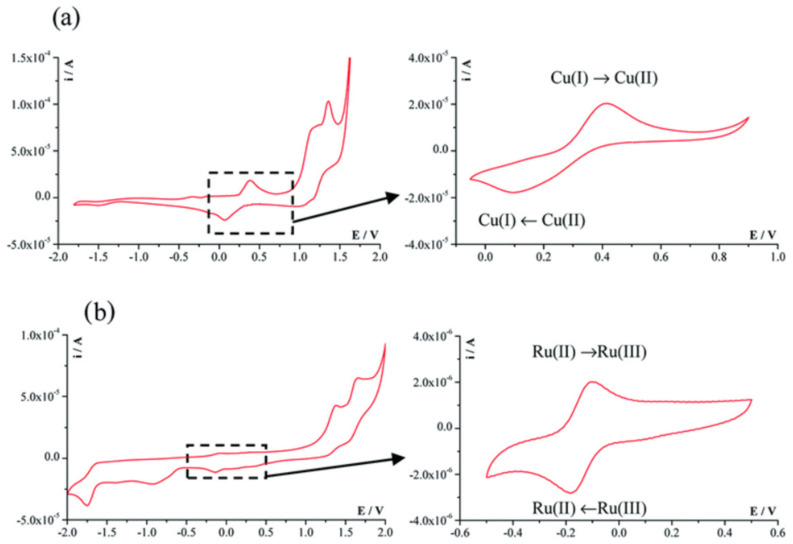
Cyclic voltammograms of the (**a**) [Cu (7 amino -flavone)Cl_2_] and (**b**) [Ru(*p*-cymene)(6 aminochromone)Cl_2_] complexes [103].

Figure 14 presents the cyclic voltammograms of the [Cu (7 amino-flavone)Cl_2_] and [Ru(*p*-cymene)(6 aminochromone)Cl_2_] complexes [103].

### 4.3. Atomic Spectroscopy

#### 4.3.1. X-ray Crystallography

X-ray crystallography gives the most concrete information about the coordination binding modes. In recent years, a lot of biological macromolecular crystallography has been performed with 3G synchrotron facilities. These facilities allowed the study of small crystals at a higher resolution because of the tunable and high-intensity X-ray sources and the fast and sensitive detectors [104,105]. The evolution of laboratory instruments, solid state detectors or CCD, and micro-source X-ray tubes has brought nucleotide oligomers (small compounds) within the range of laboratory sources [106].

Good quality data can be obtained via X-ray crystallography that provide detailed structure information at the atomic level. The method offers a 3D map of electron density from where the position of the atoms can be determined. After the position of the atoms is determined, the bond lengths and angles can be also calculated, and finally, using graphic programs, the structure can be explored and illustrated [107].

In order to use crystallography to study drug interactions with DNA, a good-quality crystal with an appropriate size is required. A considerable amount of pure and homogeneous material is needed to obtain the crystal. Also, to determine the right set of conditions, many tests are performed. This represents the limitations of the method [90].

*Small-angle X-ray scattering (SAXS)* can be used to characterize biomolecular interactions. SAXS is a high-resolution characterization method that solves features in the range of 1 to 100 nm. An advantage of using this method is the fact that biological specimens can be studied in their natural environment. In general, SAXS gives information concerning the size, shape, distributions, and locations of different nanostructures. Tagging biomolecules with high-contrast materials (for example, gold nanoparticles) determines the formation of useful molecular rulers [108]. Feigin and Svergun described the use of heavy atom labels to determine characteristic distances in different particles [109]. SAXS was used as an indication of interparticle distance for gold nanoparticles assembled in an ordered manner [110] for tumor imaging [111] and tissue characterization/differentiation [112]. Although this technique can play an essential role in the characterization of biomolecules, it also has some limitations, namely low contrast and high background noise [113]. SAXS methodology requires highly scattering molecular probes that selectively bind with high affinity to targeted biomolecules involved in putative interactions. When the targeted biomolecules come in close proximity (e.g., 1–100 nm), the probes, which are of sufficiently small size to prevent perturbing the system and avoid steric effects, provide a characteristic scattering signature that depends on intermolecular distance and is greater in intensity than that of the targeted molecules. The probes provide SAXS signatures that are indicative of the interaction between two targeted biomolecules within the specimen. SAXS methods give information regarding both interacting and non-interacting species. Other techniques, for example, positron emission tomography (PET), detect only interacting populations. The probes are designed to elastically scatter X-rays at small angles above the background signal from the biomolecules to which they are bound. SAXS gives an increased spatial resolution and a high specificity, allowing for deep tissue imaging, and complementing other methods used for biomolecular interaction detection [114].

#### 4.3.2. NMR Spectrometry

NMR studies give more information to the conformational variability in solution, thus completing the solid-state structures. The progress regarding the field strength, magnetic shielding, and cryogenic probes are advantages that the NMR spectrometry method has benefited from. For platinum anticancer agents, the most used isotopes are represented by ^1^H, ^15^N, and ^195^Pt. For speciation and kinetic studies, ^195^Pt can be used considering that it is very sensitive to the nature of the ligands attached. Sensitivity is enhanced by the use of {^1^H,^15^N} HMQC/HSQC NMR spectroscopy and is especially used in kinetic studies with biological molecules [115,116]. Magnetization transfer from the H^+^ to a second heteronuclear atom such as ^15^N gives a 2-dimensional spectrum with one axis for proton (^1^H) and another for ^15^N. So, a spectrum is formed for each H^+^ attached to ^15^N with the unique advantage of overcoming the inherent insensitivity of the ^15^N nucleus. NMR spectroscopy is widely used in practice because of its sensitivity of the chemical shift and coupling constants (e.g., ^1^J{^15^N–^195^Pt}) to the nature of the *trans* ligands, and coupled to the fact that the only H^+^ observed are those bound to the ^15^N nucleus [116,117].

The NMR spectrometry method is limited by the fact that the method needs NMR-active nucleic and mainly diamagnetic compounds [90].

#### 4.3.3. Mass Spectrometry

Electrospray Ionization Mass Spectrometry (ESI-MS) is an important method to study drug–nucleic acid interactions. It has many advantages, like the use of a small amount of sample, the speed of the analysis, and the fact that it is easy to perform. Usually, ESI-MS can be used to easily determine covalent binding with biomolecules. Also, using appropriate digestion, stoichiometry, and binding sites of metallodrugs can be determined. The non-covalent interactions receive the greatest research interest because the canonical non-covalent binding modes as electrostatic interactions, hydrogen bonding, and intercalation can be transferred to the gas phase without disruption. Thus, electrospray ionization can transfer non-covalent complexes into the gas phase of the mass spectrometer without dissociation, using appropriate non-denaturing conditions and controlled instrumental optimization. Primary spectra combined with MS-MS techniques can give information on the strength and sites of binding for single-stranded, double-stranded, and G-quadruplex DNA.

ESI-MS is used to determine the relative binding affinities of metal complexes to the duplex and quadruplex DNA. In general, the binding affinity towards quadruplex DNA is significantly less than that towards double-stranded DNA. This was demonstrated, for example, for some octahedral metallo-intercalators based on [Ru(phenanthroline)_3_]^2+^ and [Ru(phenanthroline)_2_(dppz)]^2+^ (dppz = dipyrido [3,*2*-a:*2*′,3′-c]phenazine) and on square-planar analogs such as [Pt(ethylenediamine)(phenanthroline)]^2+^ [118].

The mass spectrometry method is limited by the fact that the gas phase results may not always translate to solution [90].

*Cryo-electron microscopy (cryo-EM)* has made progress in determining macromolecular structures, especially the structures of supramolecular systems. This method presents the advantage of the possibility of studying the dynamic process and the corresponding energy change in biological samples. For the cryo-EM method, the minimum molecular weight limit of sample particles is 52 kDa [119]; the method has a good resolution, and the freezing method is more advanced. These features make the method efficient and fast, so it can compete with or replace X-ray crystallography [120]. Compared to established methods in structural biology, such as X-ray crystallography and NMR, cryo-EM has several advantages: (a) it does not need crystals; (b) it is appropriate for proteins and protein complexes with high molecular mass; (c) it reduces radiation damage and maintains the native activity and functional state of samples, including post-translational modifications; (d) in a single experiment, multiple different conformational states can be determined; (e) structural analysis of membrane proteins (for example GPCR) and their complexes can be performed; and (f) it can determine the structure of some compounds with structures that cannot be determined by X-ray crystallography. Cryo-EM limitations consist of the following: (a) if the ligands have a small molecular weight, they may not be seen in the density map; (b) the presence of organic compounds such as DMSO or glycerin in the buffer may decrease the sample contrast and resolution; and (c) the purity of the sample may be good or even very good, but the homogeneity is poor, which greatly reduces the resolution [121]. Cryo-EM has made real progress in computing image processing, such as the development of user-friendly software and the use of direct electronic detectors [122].

*Scanning electron microscopy (SEM)* provides three-dimensional images using a focused beam of electrons that scan the surface of the sample. The image is restricted to the surface of the sample, despite the high resolution (3–20 nm). This is the reason why SEM can be used in nanomedical research to characterize the spatial relationships between nanoparticles and the cell surface with particular reference to the internalization process and cell shape modification [123]. Field emission SEM (using a high-energy beam of electrons) also enables the visualization of nanoparticles in the endosomal compartment [124].

*Transmission electron microscopy (TEM)* provides images obtained by a beam of electrons transmitted through a thin specimen, thus allowing the detailed visualization of the interior of the sample. The method has a high resolution and, for this reason, can be used in nanomedical research. It is able to reveal the fine relationships between nanoparticles and cell/tissue components. To observe biological samples in TEM, it is mandatory to set up preparation procedures suitable to match the structural and/or molecular preservation with the resolution. Despite the sample processing limitations, TEM resolution remains significantly higher in comparison with light microscopy. Also, it usually permits the direct visualization of nanoconstructs and cell/tissue components without using markers [125]. TEM limitations consist of the following: (a) the observations can be made only on small and very thin sample slices (usually 70–90 nm); (b) only “static” information can be obtained due to the physical/chemical fixation and resin embedding of the sample, which excludes dynamic studies; (c) the microscope and the related equipment are more expensive than those required for light microscopy; and (d) the sample processing is time-consuming and must be performed by skilled personnel. Although it has several limitations, TEM represents an election technique to study the interactions of nanoconstructs with the biological environment [126].

### 4.4. Electrophoretic Method

#### Agarose Gel Electrophoresis

The main method to measure the nuclease activity of metal complexes is gel electrophoresis, and alone ligand shows much lower activity than a complex. Electrophoresis is an analysis method belonging to physics that is based on the migration of electrically charged particles dissolved or dispersed in an electrolyte solution and submitted to an electric field. In this process, two opposing forces determine the migration rate of the particles. One of the forces is the electrical force, which attracts the particles towards the electrodes. The electrical force is proportionate to the charge and accelerates the particles at a rate that varies directly with the charge/mass ratio. The other force is friction, which is opposed to migration, and varies with the size and form of the particle, as well as with the characteristics of the medium (viscosity, structure, etc.) [127].

The nucleic acids contain a negatively charged phosphate ion in each nucleotide. The molecular weight of the nucleotides is almost equal; thus, the charge/mass ratio is independent of the sequence and the size of the nucleic acid molecule. If the helicoidal DNA molecules are separated through electrophoresis in an aqueous solution, one can observe that the friction force opposing their migration is so small that the molecules migrate together. Moreover, they will separate through diffusion, thus hindering the analysis process. Thus, in order to separate the molecules efficiently, we need to use a medium that would greatly increase friction force and reduce the diffusion of macromolecules to a minimum. The most advantageous are gel-like supports: the polyacrylamide, the network of which is stabilized through covalent bonds, or agarose, the structure of which is formed through non-covalent interactions. If different-sized DNA molecules are separated in such a gel, one can observe that, after a certain period of time, they migrate with a mobility that is inversely proportional to log 10 of their molecular weight [128].

The nuclease activity of some metal complexes was studied by analyzing the process through which they destroy the DNA molecule (pUC18 plasmid) in the presence of reducing agents. Agarose gel was used as a migration support (porous medium), produced by dissolving 0.8% agarose in 0.5X TBE electrophoresis buffer (10 mM EDTA, 0.45 M Tris, 0.45 M boric acid) [129].

**Figure 15:**
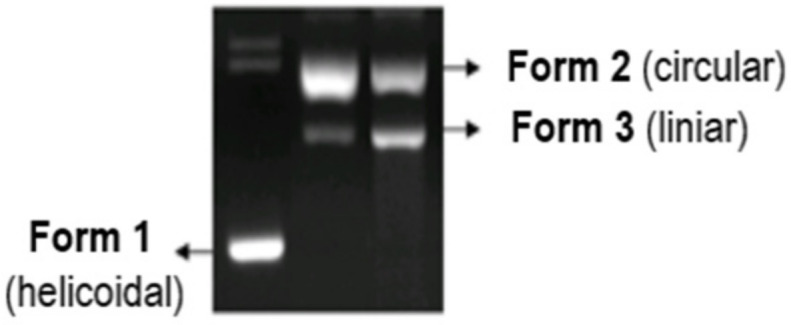
Cleavage of the DNA molecule in the electrophoresis process [34].

The studies were conducted using the pUC18 plasmid, which contains 2586 base pairs, with a 0.25 mg/mL concentration in TE buffer pH = 8.0 (Tris 10 mM, EDTA 1 mM). This plasmid is found in nature as cyclic helicoidal DNA molecules (first form). The cleavage of a chain in a single point leads to the destruction of the initial helicoidal form into a simple circular form (second form). When a chain in the double-helix is broken into two points, the result is the linear form of DNA (third form). The capacity of the synthesized complexes to destroy the DNA molecule can be determined by studying the electrophoresis behavior of the helicoidal form of DNA in agarose gel, before and after the introduction of the complex, and by studying the reducing system. The helicoidal first form migrates faster than the circular second form, while the linear third form appears after electrophoresis between the first and second forms (Figure 15) [34,130].

**Figure 16:**
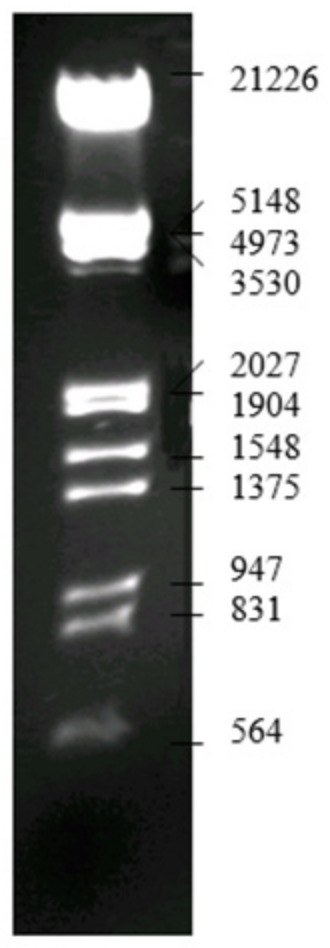
Lambda DNA/EcoRI+HindIII marker [34].

During the electrophoresis process, a base marker was used in order to determine the position of the obtained DNA fragments. The way in which each DNA fragment is represented in the electroferogram and its molar weight is presented in Figure 16.

**Figure 17:**
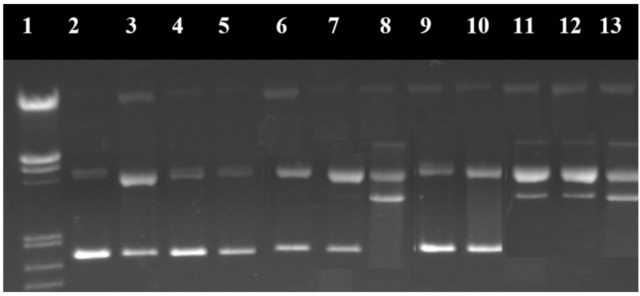
Electroferogram in agarose gel of the pUC18 plasmid treated with the [Cu(N-(5-ethyl-[1,3,4]-thiadiazole-2-yl)-toluenesulfonamidate)_2_(phenanthroline)(H_2_O)] complex. (1) Base marker; (2) control; (3) control with reducing agents; (4) CuSO_4_·5H_2_O 6 μM; (5) CuSO_4_·5H_2_O 12 μM; (6) CuSO_4_·5H_2_O 18 μM; (7) CuSO_4_·5H_2_O 24 μM; (8) CuSO_4_·5H_2_O 30 μM; (9) complex 6 μM; (10) complex 12 μM; (11) complex 18 μM; (12) complex 24 μM; and (13) complex 30 μM [131].

**Figure 18:**
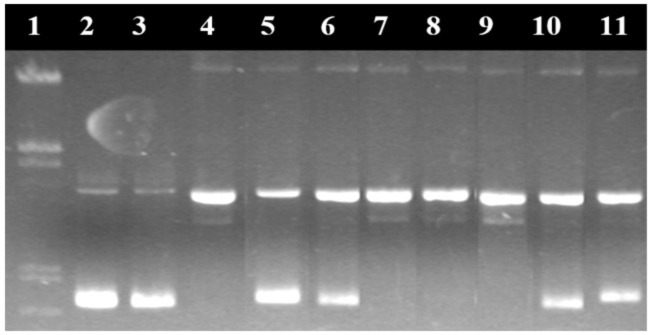
Electroferogram in agarose gel of the pUC18 plasmid treated with the [Cu(N-(5-ethyl-[1,3,4]-thiadiazole-2-yl)-toluenesulfonamidate)_2_(phenanthroline)(H_2_O)] complex and various inhibiting agents. (1) Base marker; (2) control; (3) control with reducing agents; (4) complex 15 μM without inhibitors; (5) complex 15 μM + DMSO; (6) complex 15 μM + t-butyl alcohol; (7) complex 15 μM + NaN_3_; (8) complex 15 μM + piperidone; (9) complex 15 μM + distamycin; (10) complex 15 μM + SOD; and (11) complex 15 μM + neocuproine [131].

Electroferograms in agarose gel of the pUC18 plasmid treated with the Cu^2+^ complex in the absence and presence of inhibitors are presented in Figure 17 and Figure 18 [131].

In the case of metal complexes with nuclease activity, this method was used to elucidate the reaction mechanism through which they destroy the DNA molecule by studying the free radicals and ions involved in the degradation of the nucleic acid. Thus, the nuclease activity of the complexes was studied in the presence of inhibiting agents of certain reactive oxygen species (ROS), which can capture these reactive species (e.g., dimethylsulfoxide for HO·, superoxide dismutase for O_2_^−^, sodium azide for ^1^O_2_, etc.) from their medium or which can interact with DNA molecules in another way. Dystamicin is a binder of the minor groove of DNA molecules. Neocuproine forms a very stable complex with the Cu^+^ ion that inhibits the degradation of DNA in complexes that have the reduction of the Cu^2+^ ion to Cu^+^ as a reaction step.

*Thermal denaturation studies* are also an important tool to confirm the mode of binding of metal complexes. The melting temperature of CT-DNA is influenced by the stability of the DNA double helix. The binding of complexes to CT-DNA alters the melting temperature and depends on the strength of interactions. Helix melting temperature is increased by the intercalation of small molecules into the double helix. So, the intercalation of the complexes with DNA base pairs causes stabilization of base stacking, which raises the melting temperature of the double-stranded DNA [22].

Temperature plays an important role in destroying DNA double-stranded stability. It causes the formation of the single-stranded structure or its thermal denaturation [29,132]. The melting temperature is defined to be the temperature at which 50% of DNA is denatured, with half of the nucleic acid being found in the double-stranded state and the other half in the single-stranded state [60].

The melting temperature is modified by the presence of small molecules that can stabilize or destabilize the nucleic acid structure that is directly dependent on the stability of the DNA double helix [133]. An interaction of DNA with a metal complex determines conformational changes and generally increases the melting temperature. The type and strength of interaction that takes place influence the extent of the variation in the melting temperature value.

The adenine–thymine regions of the DNA helix melt first because they contain fewer non-covalent interactions. Thus, they promote the initial unwinding of the DNA. Then, the melting of guanine–cytosine-rich regions takes place. The thermal melting process is based on the denaturation of the double helix and the loss of the secondary structure when the nucleobases become unstacked due to the loss of hydrophobic and π-π stacking interactions from the nearest neighbor. This process is reversible. Full renaturation of the DNA duplex can occur when a temperature of approximately 25 °C below the denaturation temperature is reached [134].

Many cellular processes, like transcription and recombination, are dependent on the different stability of adenine–thymine and guanine–cytosine-rich regions and on the split of the duplex into single strands. Thermal denaturation of DNA is also influenced by environmental conditions, such as the presence of salt and buffer [98].

The UV absorbance of nucleic acid is used to determine the melting temperature value and study the thermal DNA denaturation process. In the UV region (200–350 nm), the UV-vis absorption spectrum of DNA presents a broad band with a maximum of 250–280 nm. This maximum is due to the presence of chromophore groups at the level of purine and pyrimidine nucleobases, which are responsible for the electronic transitions [23,133].

When the DNA double helix is broken into two single helixes, the nucleobase interaction is diminished and the UV absorbance of the DNA solution is increased by ≈40% in comparison with the one for the DNA double helix, at the same concentration [23,29].

At low temperatures, the double-stranded form is present, while at high temperatures, the single-stranded form is present. They correspond to the maximum absorbance value.

ΔTm is defined as the difference between the melting temperature of the free DNA and the melting temperature obtained in the presence of a metal complex. Generally, a ΔTm of a few degrees Celsius is considered to be evidence of an interaction involving groove binding and/or electrostatic binding to the phosphate groups [22], while a more significant increase is attributed to an intercalation binding mode, due to a more efficient DNA double helix stabilization [133].

To estimate the strength and nature of the affinity of metal complexes towards DNA and the interactions taking place, the study of thermal denaturation represents an important tool. It is also used to determine thermodynamic parameters involved in metallodrug–DNA binding interactions that rely on the intrinsic extinction coefficient of nucleic acids [134].

The thermal denaturation method has some limitations, namely the fact that the binding information is generally provided at non-physiological temperatures (greater than 37 °C), and the stability/optical transparency of the metallodrugs is reduced at elevated temperatures [134,135].

**Figure 19:**
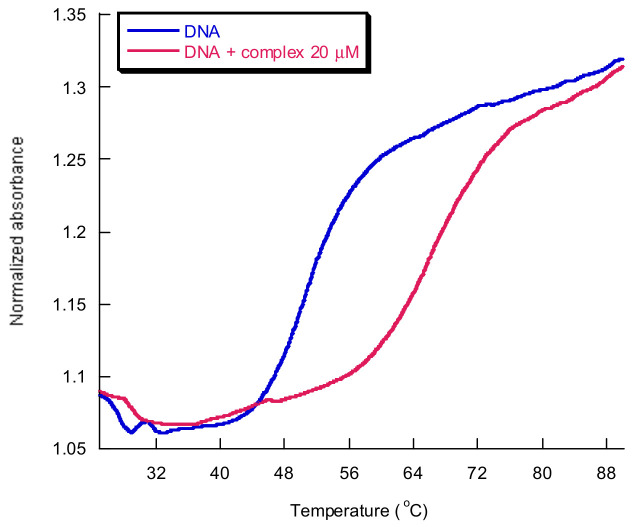
CT-DNA melting curves in the presence of 20 μM complex (red) and in its absence (blue). [Cu(N-(5-ethyl-[1,3,4]-thiadiazole-2-yl)-toluenesulfonamidate)(phenanthroline)] in cacodylate buffer 1 mM at pH 8.0, DNA:complex = 2.5:1.

An example of a melting curve for CT-DNA in the presence and absence of an assayed Cu^2+^ complex is shown in Figure 19.

*Viscosity measurement* is also an important tool to confirm DNA binding interaction. Viscosity is a direct assay method in which drug–DNA interactions are determined as a function of hydrodynamic modifications caused by the binding agent.

The viscosity of DNA increased steadily upon increasing the number of complexes added to CT-DNA, which could be compared with the classical intercalating compound ethidium bromide [136]. The CT-DNA showed a very high value of viscosity in the addition of ethidium bromide. The complexes that were found to contain aromatic chromophores showed better interaction with DNA. The extended aromatic ring of ligands partially intercalated into the DNA base pairs and the higher hydrophobicity of the aromatic ligand elongates the DNA chain. However, the incorporation of steric hindrance in the ligand prevents partial intercalation and leads to the lengthening of DNA. A classical intercalation model usually results in lengthening the DNA helix. In this case, the base pairs are separated to accommodate the binding ligand, and this leads to an increase in DNA viscosity [137].

This technique is sensitive to changes in DNA chain length and considering that covalent and non-covalent binding modes display different hydrodynamic characteristics, individual modes of binding can be distinguished. To separate the base pairs to accommodate the bound ligand, intercalators determine the extension and unwinding of the DNA backbone. This results in the lengthening of the DNA molecule in proportion to the amount of drug bound. In the case of non-covalent major and minor groove binding agents, the opposite effect can be found because it causes little or no distortion to the phosphate backbone of DNA [138].

**Figure 20:**
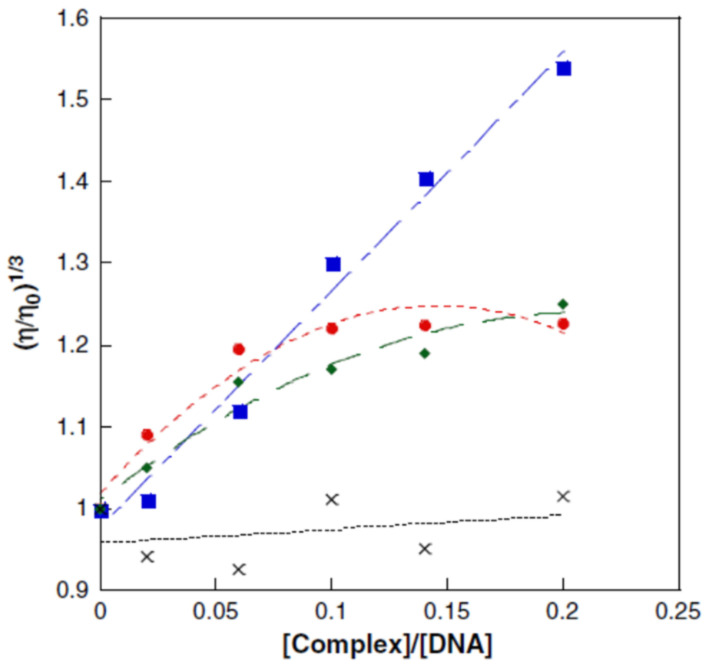
The influence of Cu^2+^ complexes on the viscosity of a DNA solution: blue—[Cu(NST)_2_(phenanthroline)]: intercalation; red—[Cu(NST)_2_(NH_3_)_2_]·H_2_O: minor/major groove interactions; green—[Cu(phenanthroline)_2_]^2+^: partial intercalation; and black—CuCl_2_: standard NST = *N*-2-(4,5-dimetylthyazol)naphtalenesulfonamide [139].

The viscosity measurement is limited by the fact that the compounds must result in changes in the viscosity of the DNA solution and that metal can compact DNA by charge neutralization [98]. Figure 20 presents the influence of some Cu^2+^ complexes on the viscosity of a DNA solution [139].

Experimental techniques and analysis used for routine DNA studies can be extrapolated to coordination complexes in most cases and this is also true with **Computational Chemistry tools**, except for the fact that transition metals are still complex to simulate due to their multiple oxidation states and resulting geometry change; therefore, special consideration should be given when generating force field parameters or applying a quantum mechanical method to these molecules [140].

*Molecular dynamics* studies provide information concerning the general structure of the binding mechanism. Even if no electronic effect is taken into account, these simulations give information regarding the dynamics where the metal compound is binding and also about the conformational changes that take place in the whole system. A simulation of a B-DNA stable chain in the microsecond realm and realistic sampling of the global and local parameters is carried out using the AMBER and the CHARMM family of force fields for nucleic acids [141]. Based on NMR spectra, molecular dynamics allows refinement based on the Nuclear Overhauser Effect restraints of distance, angle, and penalty functions. These analysis tools represent an efficient way to study the structure of a metal compound and to determine the way it binds to nucleic acids. The problem is the fact that it requires the restrained information obtained by NMR spectroscopy [142]. General force fields do not have the parameters to describe a bioinorganic compound that contains a transition metal. This represents a concern that has to be resolved with the aim of being able to run a molecular dynamic simulation. The Metal Center Parameter Builder, which is available in the Amber programs [143], represents a possibility to generate the bonded and non-bonded parameters around a metal center. It is easier to generate the required force field parameters using the bond, angle, and dihedral values if the X-ray or NMR structure of the metal compound is determined. For some metals, non-bonded parameters are available. The resulting force field values can be used to recreate the metal compound and run molecular dynamics for several nanoseconds. Thus, its stability and correct representation for the simulation is assured [144].

If no experimental structure is available, *quantum mechanical methods* may be used to optimize an initial structure. Using this structure, the appropriate force field parameters can be generated. The use of quantum mechanical methods allows a full electronic study of the system and a considerable reduction in empirical approximations. These methods are extremely computational and memory-demanding, and the representation of solvent interactions is limited. With current methods, explicit solvent representation is not reachable. It can be used as a generated molecular dynamics simulation with explicit solvent and transfer specific water molecules for quantum mechanical treatment. The computing time is long because transition metals contain a large number of electrons and it is important to study every electron of the metal atom. However, in most cases, the core electrons are not used for reactions or energetics. This can be simulated using effective core potentials which “freeze” core electrons and facilitate the electronic representation of the metal, requiring less time to achieve conversion. A simple approach to studying these interactions is the use of isolated bases and nucleotides, which basically strip apart the DNA or RNA chain. This allows the use of high-level computational and analysis methods. Another advantage of quantum mechanical methods is represented by the possibility of obtaining spectroscopic data (UV-vis, fluorescence, and/or RAMAN). This provides a way to compare experiments and simulations, starting with specific configurations [145].

In conclusion, Table 2 presents the analytical methods used for the determination of metal-based drug–DNA interactions with the main advantages and limitations.

## 5. Conclusions

The therapeutic potential of metal complexes in cancer therapy is of particular research interest, as metals present several characteristics, such as redox activity, different coordination modes, and reactivity towards the organic substrate, which is essential in the design of antitumor agents. They must be able to selectively interact with the biomolecular target and subsequently alter the cellular mechanism of proliferation. The development of effective antitumor drugs with high selectivity and low toxicity is currently a major challenge for the scientific community. Therefore, the evaluation of potential metal-based drug-binding properties for DNA interaction is a crucial step toward identifying the ultimate targets of compounds and consequently towards understanding their modes of action. From the examination of the recent literature, as demonstrated above, the reader can gain an appreciation that there is an armory of analytical techniques to examine different aspects of interactions of metal-based drugs with DNA, such as adduct formation, conformational changes in DNA, DNA degradation, etc. Once the types of complex metallic–DNA molecule interactions were established, research evolved to identify the most complete and complex methods to identify these modes of metal drug–DNA interactions. Thus, the literature abounds in information about qualitative or quantitative analytical methods, classic or modern, and more accessible or less accessible, in an attempt to establish as precisely as possible the mode of interaction/binding of metal complexes with DNA. Most of the time, it is necessary to use several complementary methods in order to obtain the most accurate information. Some of these methods are computational, so they cannot be employed in physiological conditions; in other situations, the structure of the metal drug does not allow the correct identification of the binding points with DNA and, therefore, additional determinations are needed.

Our review provides a synthesis of the methods for determining the types of complex metallic–DNA interactions currently used, specifying the advantages and disadvantages in each case. In our opinion, in approaching this complex subject it is necessary to unravel the mechanisms through which the drug is metabolized in the bloodstream, delivered to the cell, inserted into it, and processed until it comes into contact with the DNA. Likewise, the identification of all relevant metal species should be addressed through the use of integrated separation and detection techniques as well as metallogenomics procedures. For preliminary studies, the use of the presented methods can be a step forward in the identification of new metal compounds with therapeutic properties.

## Figures and Tables

**Table 1:** Examples of metal complex–DNA interactions.

Metal Complexes Investigated for Interaction with DNA		DNA Type	Identified Interaction	Reference
Metal Ion	Ligand Type			
Copper (II) Platinum (II)	2-((2-(pyridin-2-yl)-1H-benzo[d]imidazol- 1-yl)methyl)quinolone	ct-DNA and pBR322 plasmid DNA	Intercalation	Li et al. [81]
Cobalt (II) Nickel (II) Copper (II)	5-methyl-2-phenyl-1,2-dihydro-3Hpyrazole-3-one and 3-methyl-1-phenyl4-[(E)- phenyldiazenyl]−4,5-dihydro-1H-pyrazole5-ol	ct-DNA and pUC-19 DNA	Intercalation	Kirthan et al. [82]
Iron (III)	1-amino pyrene and 2- hydroxy-1-napthaldehyde	ct-DNA	Intercalation	Saha et al. [83]
Palladium (II) Vanadium (II) Silver (I)	1-(Pyridin3-yliminomethyl)-naphthalen-2-ol (HNAP)	ct-DNA	Intercalation	Abu-Dief et al. [84]
Rh (III)	2,2-bypiridine, 5,6-chrysenequinone diimine 2,2-bipyridine, dipyridophenazine	pUC-19 DNA	Insertion	Exleben [67]
Copper (II) Iron (III) Palladium (II)	Schiff bases of 2-hydroxy-1-naphthaldehyde and Schiff bases of 4-amino-acetophenone	PcDNA3.1 (-) plasmid DNA	Groove binding	Kurt et al. [85]
Copper (II)	Esculetin	ct-DNA	Minor groove binding	Shinde et al. [86]
Cobalt (II) Nickel (II) Copper (II)	2-(4-sulfametazin)hidrazono-5,5dimetilsikloheksan-1,3-dion	ct-DNA	Electrostatic interaction or groove binding	Kiwaan et al. [87]
Copper (II)	1-methyl-l-tryptophan	pBR322 plasmid DNA and ct-DNA	Intercalation or electrostatic interaction	Baskaran et al. [88]

**Table 2:** Comparison of different analytical techniques for studying metallodrug–DNA interactions.

Type of Method	Main Advantages	Main Limitations
**Fluorescence spectroscopy**	- Evaluation of the affinity of metal complexes towards DNA. - High sensitivity. - Strong selectivity. - Small sample volume.	- Insufficient application range. - Sensitivity to the environment. - Indirect methods with a reliance on the signal from a fluorogenic reporter.
**Cyclic voltammetry**	- Determination of metal complex–DNA interaction mechanisms. - Evaluation of drug–DNA binding parameters. - The reverse sweep provides additional information to help identify materials. - Converts material back into its original form, which can prevent the accumulation of unwanted material.	- The effect of slow heterogeneous electron transfer.
**X-ray crystallography**	- Gives the most concrete information about the coordination binding modes. - Provides detailed structure information at the atomic level. - Enables the study of small crystals at a higher resolution.	- A good quality crystal with an appropriate size is required. - A considerable amount of pure and homogeneous material is needed.
**Small-angle X-ray scattering**	- Characterization of biomolecular interactions. - Biological specimens can be studied in their natural environment. - Deep tissue imaging/tumor imaging. - Tissue characterization/differentiation. - High resolution and specificity.	- Low contrast. - High background noise.
**NMR spectrometry**	- Provides more information about the conformational variability in solution. - Obtains accurate three-dimensional structural information from molecular vibrations within the natural environment, keeping the sample intact.	- Needs NMR active nucleic and mainly diamagnetic compounds. - Low sensitivity of NMR instruments to insufficient sample concentrations.
**Mass spectrometry**	- Studies drug–nucleic acid interactions. - The use of a small amount of sample. - The speed of the analysis.	- The gas phase results may not always translate to a solution.
**Cryo-electron** **microscopy**	- Determination of the macromolecular structure. - Structure determination of some compounds whose structures could not be determined by X-ray crystallography. - Studies the dynamic process in the case of biological samples. - Does not need crystals.	- The small molecular weight of the ligand. - The presence of organic compounds such as DMSO or glycerin in the buffer. - The poor homogeneity of the sample.
**Scanning electron microscopy**	- Can be used in nanomedical research. - Characterizes the relationship between nanoparticles and the cell surface.	- The image is restricted to the surface of the sample.
**Transmission** **electron microscopy**	- Can be used in nanomedical research. - Reveals the relationships between nanoparticles and cell/tissue components.	- Requires small and very thin sample slices. - Excludes dynamic studies.
**Agarose gel electrophoresis**	- Determination of the nuclease activity of some metal complexes. Analyzes the process/mechanism of action through which metal complexes destroy the DNA molecule.	- Limited accuracy and sensitivity. - Time-consuming process. - Lack of real-world applications.
**Thermal denaturation**	- Estimates the strength and nature of the affinity of metal complexes towards DNA and the interactions taking place.	- Sensitivity to the environment. - Binding information is generally provided at non-physiological temperatures (greater than 37 °C). - Less stability/optical transparency of the metallodrugs.
**Viscosity measurement**	- Important tool to confirm the DNA binding interaction. - Provides accurate and repeatable measurements. - Single or multiple measuring points.	- The compounds must result in changes to the viscosity of the DNA solution and the metal can compact DNA by charge neutralization.
**Molecular dynamics studies**	Studies the structure of a metal compound and determines the way it binds to nucleic acids.	It requires the information obtained by NMR spectroscopy.
**Quantum mechanical** **methods**	- Optimization of an initial structure. - Possibility to obtain spectroscopic data.	- The computing time is long. - The representation of interactions with solvent is limited.
